# Concept and Realization of a Novel Test Method Using a Dynamic Test Stand for Detecting Persons by Sensor Systems on Autonomous Agricultural Robotics

**DOI:** 10.3390/s21072315

**Published:** 2021-03-26

**Authors:** Christian Meltebrink, Tom Ströer, Benjamin Wegmann, Cornelia Weltzien, Arno Ruckelshausen

**Affiliations:** 1 Faculty of Engineering and Computer Science, University of Applied Sciences Osnabrück, 49076 Osnabrück, Germany; tom.stroeer@hs-osnabrueck.de (T.S.); a.ruckelshausen@hs-osnabrueck.de (A.R.); 2 Agromechatronic, Technische Universität Berlin, 10623 Berlin, Germany; cornelia.weltzien@tu-berlin.de; 3 B. Strautmann & Söhne GmbH u. Co. KG, 49196 Bad Laer, Germany; B.Wegmann@strautmann.com; 4 Leibniz Institute for Agricultural Engineering and Bioeconomy (ATB), 14469 Potsdam, Germany

**Keywords:** image-based sensor systems, object detection systems, safety of autonomous agriculture machines, test stand, humanoid test target

## Abstract

As an essential part for the development of autonomous agricultural robotics, the functional safety of autonomous agricultural machines is largely based on the functionality and robustness of non-contact sensor systems for human protection. This article presents a new step in the development of autonomous agricultural machine with a concept and the realization of a novel test method using a dynamic test stand on an agricultural farm in outdoor areas. With this test method, commercially available sensor systems are tested in a long-term test around the clock for 365 days a year and 24 h a day on a dynamic test stand in continuous outdoor use. A test over a longer period of time is needed to test as much as possible all occurring environmental conditions. This test is determined by the naturally occurring environmental conditions. This fact corresponds to the reality of unpredictable/determinable environmental conditions in the field and makes the test method and test stand so unique. The focus of the developed test methods is on creating own real environment detection areas (REDAs) for each sensor system, which can be used to compare and evaluate the autonomous human detection of the sensor systems for the functional safety of autonomous agricultural robots with a humanoid test target. Sensor manufacturers from industry and the automotive sector provide their sensor systems to have their sensors tested in cooperation with the TÜV.

## 1. Introduction

Work on the development of autonomous machines has been going on for years. In the agricultural sector, technical solutions for autonomous machines have already been developed. These solutions range from autonomous feeding mixer [1], to autonomous field robots [2], to already 20-year-old autonomous tractors [3]. In addition to the technical realization of autonomous functions, a key technology is the functional safety of autonomous agricultural machinery. It depends largely on the functionality and robustness of non-contact sensor systems for human protection. Solutions that have already established themselves in the industrial environment meet new challenges in the agricultural sector and demand a new approach.

In a final thesis, first approaches for test scenarios for the validation of autonomous field robots with non-contact sensor systems were developed in 2015 [4]. Tiusanen et al. [5] give an overview of the current state of safety requirements for autonomous machines and show three different approaches for a safety concept. Basu et al. [6] shed light directly on the legal situation for the operation of small agricultural robots and Ingibergsson [7] developed a rule-based language to force safety requirements on cameras and computer vision. International expert groups, e.g., European Agricultural Machinery Association (CEMA), are working on specifications for agricultural machinery manufacturers for the selection of non-contact sensor technology for human protection and the validation of autonomous agricultural machinery. Despite these activities, the demand of non-contact sensor technology, which is approved for outdoor human protection, increases. The IEC 62998-1:2019 [8] defines specifications for the development and evaluation of safety-relevant sensors for the protection of persons in outdoor areas. It defines an assistance to evaluate and develop sensor systems individually for the planned field of application. Jakobs et al. [9] proposes a concrete procedure based on the standard and the integration into the overall development process.

For the required proof of the detection capability of non-contact sensor systems in outdoor areas, a concept and the realization of a novel test method is presented in this article and implemented by a test stand for sensor systems. With this test method, commercially available sensor systems are tested in a long-term test around the clock for 365 days a year and 24 h a day on a dynamic test stand in continuous outdoor use. A test over a longer period of time is needed to test as much as possible all occurring environmental conditions. This leads to the fact that it is a test that is determined by the naturally occurring environmental conditions and therefore cannot be planned. This corresponds to the reality of unpredictable/determinable environmental conditions in the field and makes the test method and test stand so unique. The focus of the developed test methods is on testing the autonomous human detection of the sensor systems for the functional safety of autonomous agricultural robots with a humaniod test target.

**Figure 1:**
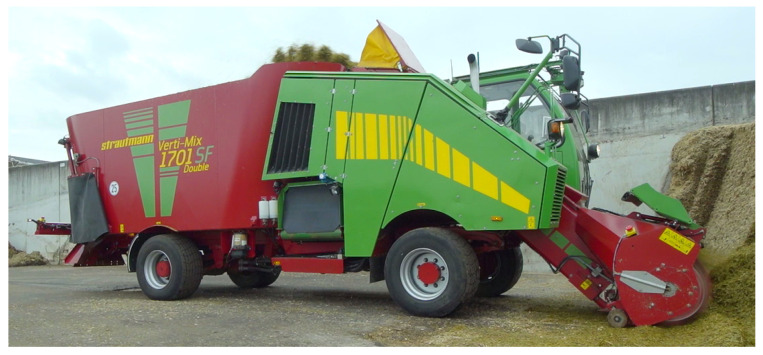
An unmanned feeding mixer is already working with autonomous driving and working functionality. In the developed safety concept, a non-contact sensor system is still missing for the human protection at the autonomous feeding mixer, which may be used both for indoor and outdoor on an agricultural environment.

Since the verification of safe human detection for the functional safety of autonomous agricultural machinery is not universally possible due to the variety of application areas and environmental conditions, the first test stand has been implemented for a specific application example. Here, the autonomous feeding mixer [1] of the company B. Strautmann und Söhne GmbH & Co. KG is used, as it is one of the agricultural robots that is furthest along the road to market, has defined operating limits and operates at low speeds (max. 2 m/s). In Figure 1, the feeding mixer is shown in autonomous operation. The technical realization is largely completed and a new safety concept is developed in cooperation with the TÜV. In order to achieve the set safety goals, a non-contact sensor system is required, which reliably detects humans outside at an early stage, that the autonomous machine reaches a safe state.

For this reason, an individual test stand is developed for the autonomous feed mixer using the new test methods at the University of Applied Sciences Osnabrück in cooperation with the company B. Strautmann und Söhne GmbH & Co. KG, the TÜV and the Technische Universität Berlin in the research project “Agro-Safety”, funded by the BMBF and B. Strautmann und Söhne GmbH & Co. KG. With this dynamic test stand, commercially available sensor systems are tested in a long-term test around the clock for 356 days a year and 24 h a day in continuous outdoor use and a system will be selected that meets the individual requirements from the specific environmental conditions and machine parameters. If one sensor system alone does not meet the requirements, a combination of several sensor systems is possible. This combination can be realized on the basis of different fusion options.

## 2. Concept of a Test Stand

As an impact of research into industry, a dynamic test stand has been developed and realized on an agricultural farm for the very first time. Commercially available sensor systems are tested in a long-term test around the clock for 365 days a year and 24 h a day on a dynamic test stand in continuous outdoor use. A test over a longer period of time is needed to test as much as possible all occurring environmental conditions. This leads to the fact that it is a test that is determined by the naturally occurring environmental conditions and therefore cannot be planned. This corresponds to the reality of unpredictable/determinable environmental conditions in the field and makes the test method and test stand so unique. Due to the wide variety of environmental conditions, a sensor system will always need to be tested individually for the specific location of its autonomous robot. For this reason, the test stand is located between a silo installation and a cultivated agricultural area. Thus, the sensor systems are exposed to the general environmental conditions in the outdoor area, the environmental conditions in a silo plant and a cultivated field. In the silo, various particles of different sizes can be present in the air during silaging or feed intake, which can disturb the sensor systems. Dust formation during the summer months is, among other things, an interesting factor for sensor systems on a cultivated, agricultural field.

### 2.1. The Sensors

Eight different sensor manufacturers from industry and the automotive sector provide a total of 15 sensor systems with six different measurement principles for the test stand.

In this article, a sensor system is understood as the combination of a measurement unit, the pure sensor, and the measurement data interpretation. The sensor can be realized by different measurement principles (e.g., LiDAR, radar, etc.) and generates measurement principle dependent raw data. In the measurement data interpretation, the raw data are interpreted and a decision is created, if an object is detected or not. For this reason, the term “object detection system (ODS)” is introduced at this point of the article and used instead of “sensor system”. In the following, an ODS is understood as a sensor with an evaluation unit.

The 15 ODSs are composed of the following measurement principles:

- Three single-line LiDAR sensors with outdoor or safety features.
- One multi-line LiDAR sensor.
- One ToF camera.
- One stereo camera.
- Three radar sensors with 24 GHz or 77 GHz.
- Six ultrasonic sensors, two from each manufacturer with different detection ranges.

Thus, six different sensor types are tested simultaneously on the test stand and are compared with each other under different environmental conditions. It is important that three groups are formed and are not tested simultaneously but one after the other. Each group is assigned only to ODS with measuring principles that cannot influence each other. Due to the same measuring principles, the ODS can interfere with each other or with one another. The ODSs are optimally adjusted to the expected environmental conditions on site at the test stand by the respective manufacturers. Thus, a manufacturer independent test of the ODS can be guaranteed, without setting errors due to ignorance can influence the test result.

### 2.2. The Test Target

**Figure 2:**
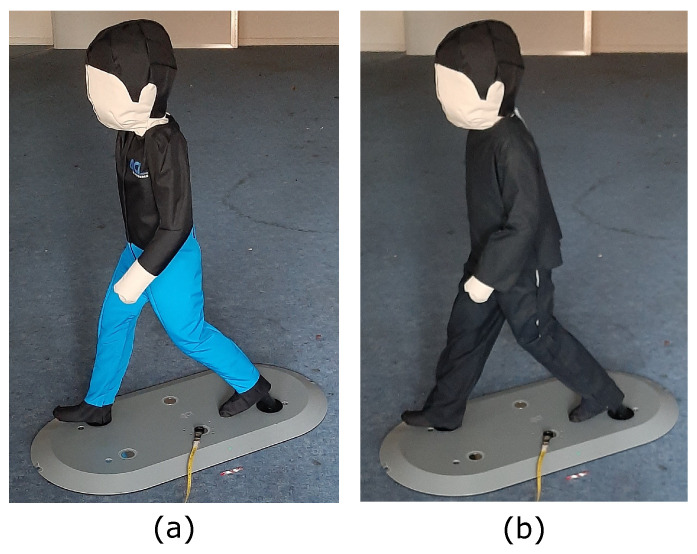
(**a**) Test target “4activePS child (v3v3.2)”; (**b**) modified test target with cotton.

As a basis, the test target “4activePS child (v3v3.2)” of the company 4activeSystems GmbH from Traboch in Austria [10] is used, which is shown in Figure 2a. It represents a 6–7 year old child, is used in the Euro NCAP test for pedestrian emergency brake assistants [11] and defined in the standard ISO 19206-2:2018-12 [12]. In the publication “Evaluation of Pedestrian Targets Used in AEB Testing: A Report from Harmonistion Platform 2 Dealing with Test Equipment” [13], its properties as a test target are presented and discussed. It is supposed to be a technology-independent test target, which after various tests reflects all relevant physical properties for the most common sensors. According to the manufacturer’s statement, the test target’s properties are not “worst-case” parameters of any technology, since the test target is only used to test driver assistance systems. The new test stand is intended to test ODS for use on unmanned machines and vehicles. For this reason, certain properties of the test target are adapted accordingly in the research project. In Figure 2b, the modified test target is shown.

**Figure 3:**
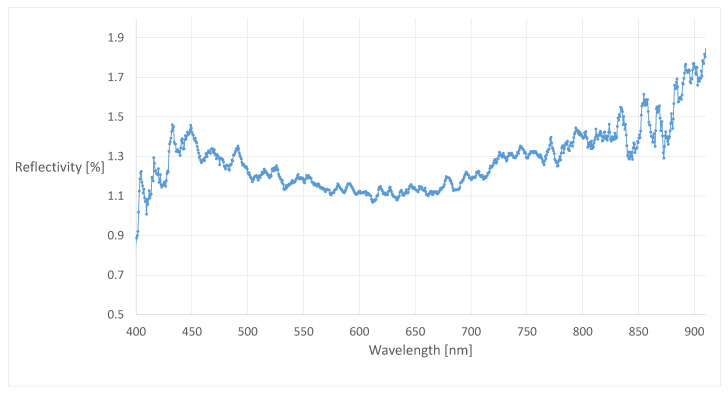
Surface reflectivity of the new cotton dress in the wavelength range from 400 nm to 910 nm.

For ODS with a optical sensor, the standard ISO 19206-2:2018-12 [12] specifies clothing and visible skin with reflectance levels between 40% and 60% for the near-infrared (NIR) wavelengths from 850 nm to 910 nm for the manufacturer test target. The hair should have a degree of reflection of 20–60% in this wavelength range. If these values are compared, for example, with specifications from the standard ISO 3691-4:2020-02 [14] for industrial trucks, it becomes clear why the manufacturer points out that this is only a test target for driver assistance systems. The draft standard requires a test target with a surface reflectance of 2–6% depending on the location of the vehicle for the validation of unmanned systems. Discussions with manufacturers of safety sensor systems for industrial trucks have confirmed that a surface reflectance of 5% will be tested and validated. For this reason, the previously described test target receives new clothing that guarantees a surface reflectance of 2–6% for the corresponding wavelengths for the optical ODS. This surface reflectance is achieved with a black, outdoor-suitable cotton. The standard ISO 19206-2:2018-12 [12] specifies a measurement of the degree of reflection at a defined angle (90° and 45°). In this project, it was limited to perform all subsequent measurements with an angle of 90°. In Figure 3, the surface reflectivity of cotton in the wavelength range from 400 nm to 910 nm is shown, which included the visible light range (400–780 nm) and the NIR wavelength range (850–910 nm).

It can be seen in Figure 3 that the new cotton has a reflectivity between 1.29% and 1.85% in the wavelength range from 850 nm to 910 nm and falls below the normative range of 2–6%. Since a deterioration of the reflectivity is expected during permanent outdoor use, the low reflectivity is considered suitable. For systems, e.g., cameras, which do not only work in the NIR wavelength range, the wavelength range between about 400 nm and 780 nm in the visible light range is also presented in Figure 3. For this wavelength range, the new cotton has a reflectivity between 0.89% and 1.46%. The aging of the materials during outdoor use is checked and taken into account by spectral measurements.

In a project of the University of Applied Sciences Osnabrück [15], the radar reflectivity of a 24 GHz sensor war measured and verified with the data of the test target manufacturer. Comparative measurements with real people were also carried out. It was shown that the reflectivity of the test target is lower than that of an average 28-year-old male person. By comparing further reflection measurements with the manufacturer’s data, the study concludes that the reflection properties of an average person can be reproduced. The body height of the test target is significantly smaller compared to the male test person and in this ratio a lower reflection was also measured. For this reason, it is assumed that smaller persons such as children have a similar reflection to the test target. In addition, the project work [15] investigated the effect of clothing on the radar reflectivity of the test target. As described in the publication [13], it was confirmed that clothing has a negligible effect on the reflective properties of a person or test target. Thus, it could be demonstrated that the new cotton clothing has no significant effect on the reflective properties of the test target.

The ultrasonic reflective properties of the test target are considered realistic and are left unchanged by the clothing and round shapes of the test target to simulate people.

### 2.3. Speed Definition

All ODSs are moved simultaneously within a defined movement space. The ODS and the target are accelerated with 8 $\frac{m}{s^{2}}$. The ODS is moved at a speed of 2 $\frac{m}{s}$. This speed corresponds to the maximum speed of the autonomous feeding mixer.

In the Euro NCAP test for testing pedestrian emergency brake assistants, the maximum tested speed of pedestrians is 8 $\frac{\mathrm{km}}{h}$ (approximately 2.22 $\frac{m}{s}$). This speed simulates a running adult pedestrian [11]. A child pedestrian running onto the street is simulated with 5 $\frac{\mathrm{km}}{h}$ (approx. 1.38 $\frac{m}{s}$) [11]. The standard ISO 19237:2017-12 [16] for intelligent transport systems define as well a pedestrian speed of 5 $\frac{\mathrm{km}}{h}$ (approximately 1.38 $\frac{m}{s}$). If speed values from other sources are compared with these values, it can be assumed that these values are average speeds. Bartels et al. [17] have compared different sources with pedestrian speeds in their publication. According to Bartels et al. [17] their sources all define similar values. According to the source they cited, Kramer et al. [18], men at the age of 35 move fastest at 6.78 $\frac{m}{s}$. This speed should correspond to a race without an acceleration phase. Children at the age of 5 years run without an acceleration phase according to Kramer et al. [18] a maximum of 3.51 $\frac{m}{s}$ (male) and 3.49 $\frac{m}{s}$ (female).

A test target velocity of 2.3 $\frac{m}{s}$ is used in this project. This value corresponds approximately to the value of 2.22 $\frac{m}{s}$ which is defined in the Euro NCAP tests [11] which is mentioned above. As described, this speed corresponds to the maximum tested speed and is used for jogging, adult persons. The test target simulates a running child aged 6–7 years according to the ISO 19206-2:2018-12 standard [12]. This child is simulated with approximately 1.38 $\frac{m}{s}$ for the Euro NCAP test [11]. Summarizing the defined speed of 2.3 $\frac{m}{s}$ does not reach the maximum speed of 3.51 $\frac{m}{s}$ from Kramer et al. [18], but still corresponds to over 80% of pedestrian speeds [19] according to a graph of a study by the Japanese Society of Automotive Engineers (JSAE). Additionally, the target speed corresponds approximately to the maximum tested speed of the Euro NCAP tests [11]. Even higher speeds from other sources were not considered because faster objects would have to be detected further ahead in order to be able to react to them early. This early detection of objects is not simulated and will be tested on this test stand. An overview of pedestrian speeds and the classification of the applied test target speed are shown in Table 1.

### 2.4. Technical Setup

For a better understanding of the following test methods, this section describes the basic technical concept of a test stand that can be used to implement the test methods for a specific application example. The described dimensions and technical parameters of the concept below can be varied and adapted depending on the application example. In the following, the basic technical concept is adapted for our application example, the autonomous feeding mixer.

**Figure 4:**
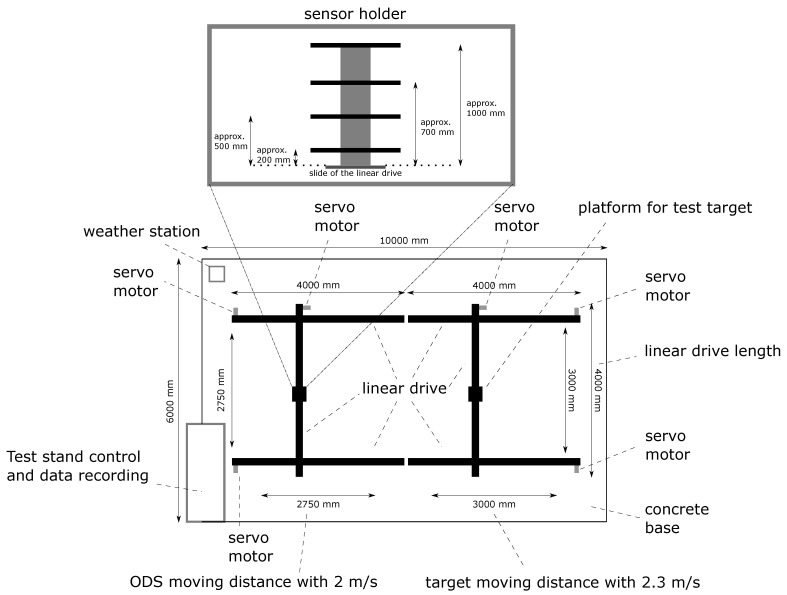
Top view in a schematic drawing of the test stand is shown and gives an overview of the dimensions of the test stand and the traverses of the object detection system (ODS) and target. The structure of the sensor holder is also shown.

The test stand consists of a movement space for the ODS and a movement space for a test target, which must be recognized by the ODS. This movement space is realized by means of two two-axis gantries, each with an area of 4 m length and 4 m width on a concrete base of 10 m length and 6 m width in total. With the consideration of safety distances, a field of 3 m length and 3 m width remains for the ODS. Taking acceleration and braking distances into account, a travel distance with constant speed of 2 $\frac{m}{s}$ of 2.75 m length and 2.75 m width remains. The ODSs are attached to a sensor holder that is positioned vertically upwards. On this sensor holder, there is the possibility to mount the ODS at four different heights. In this way, different mounting positions can be realized on a mobile machine. For better comparability of identical measuring principles, care is taken to ensure that this ODS are at the same height. Figure 4 shows a schematic drawing of the test stand. The drawn length ratios are not shown in reality.

As shown in Figure 4, with the second two-axis gantry, a field of 3.25 m length and 3.25 m width is realized for the test target, taking into account safety clearances, as a range of motion. Taking acceleration and braking distances into account, a travel distance with constant speed of 2.3 $\frac{m}{s}$ of 3 m length and 3 m width remains.

With the previously shown values, a specified detection area (SDA) of up to 5.75 m length and 5.5 m width could be tested with such a test stand. The SDA is a region of interest which can be defined for the ODS. The object detection systems can focused on the object detection on this area. The length of 5.75 m results from the addition of the travel distances of the ODS of 2.75 m and the test target of 3 m when the ODS is aligned in the direction of the test target. For ODS with a wide detection range, the test stand can test the left and right side of the specified detection field of the ODS separately. For this reason, the total width of 5.5 m results from the double travel of the ODS of 2.75 m.

At the upper left corner of the concrete base in Figure 4, a weather station and a visibility measuring device is placed. The test stand control with the data recording is located at the lower left edge of the concrete base.

### 2.5. The Test Method

The core of the test stand is a novel test method with different test scenarios. This test method creates real environment detection areas (REDAs), which can be used to compare different ODSs with different measurement principles in different environmental conditions.

To record the REDA in the detection range of the ODS, the test target is moved through the specified detection area (SDA) from different directions on the basis of different test scenarios. The test stand can evaluate at which points the test target is detected by the ODS under the current environmental conditions. The SDAs are defined by the manufacturers before the test will be started.

In the following section, the individual test scenarios of the test method are presented. Then, in Section 2.5.2,the formation of REDA is explained on the basis of the test scenarios. Section 2.5.3 describes the creation of REDAMs based on the REDAs and the consideration of the different environmental conditions. In Section 2.5.4, the evaluation of the REDAs and REDAMs is explained.

#### 2.5.1. Test Scenarios

The test method consists of different test scenarios to create the REDAs. The test scenarios represent different states of the ODS and the test target to determine the corresponding REDAs under the different environmental conditions. A distinction is made between the states “static” and “dynamic”. The “static” state describes a stationary position, whereas the “dynamic” state represents a movement. This results in four different categories of test scenarios:

1. Category 1, static ODS, static test target: In this category, the ODS and the test target are in a fixed position. This category represents, for example, the scenario where an autonomous agricultural machine is in park position and a person is standing directly in front of the machine. It can be checked whether the ODS detects the person in this situation at an early stage and thus no dangerous situation arises, for example, when the machine is started up.
2. Category 2, static ODS, dynamic test target: The second category describes a stationary ODS and a dynamic test target. This category is used to systematically cover the SDA of an ODS in the idle state. As a real situation, a person could run in front of the agricultural machine shortly before starting up. The ODS must detect the person and prevent the machine from starting up.
3. Category 3, dynamic ODS, static test target: In the third category, the ODS is moved and the test target is stationary. This category describes a standing person in front of a moving autonomous agricultural machine.
4. Category 4, dynamic ODS, dynamic test target: The fourth category describes a moving ODS and a moving test target. It simulates a moving autonomous agricultural machine and a person running into the roadway.

On the test stand, the ODSs are to be tested in the two states, “static” and “dynamic”, under different environmental conditions and the corresponding REDAs are to be determined. Thus, the following 5 questions arose, which have to be answered on the basis of the tests:

1. Can the ODS detect the test target under the current environmental conditions?
2. How large is the REDA of the ODS in static state under the current environmental conditions?
3. Are there gaps and detection faults in the REDA of the ODS in the static state under the current environmental conditions?
4. How large is the REDA of the ODS in dynamic state under the current environmental conditions?
5. Are there gaps and detection faults in the REDA of the ODS in the dynamic state under the current environmental conditions?

The questions listed are intended to provide a quantitative evaluation of the test stand and its test methods. The answers are given in Section 3.

The test categories will be realized by one or more test scenarios. Each test scenario will be performed one after the other. Test scenario category 3 describes a real state at the autonomous agricultural machine. Due to the higher relative speeds between the ODS and the test target in test category 4, test category 3 is not used in the tests. It is also a balance between all possible test scenarios and the changing environmental conditions. If the tests take too long, the risk of not testing all scenarios under the same environmental conditions increases. As a first approach, this test method is limited to the categories 1, 2 and 4 described above. In a later validation on the autonomous feeding mixer, further test categories have to be considered and proven. In the following figures, red arrows indicate the movements during a test recording is performed. Black arrows indicate movements without test recording.

**Figure 5:**
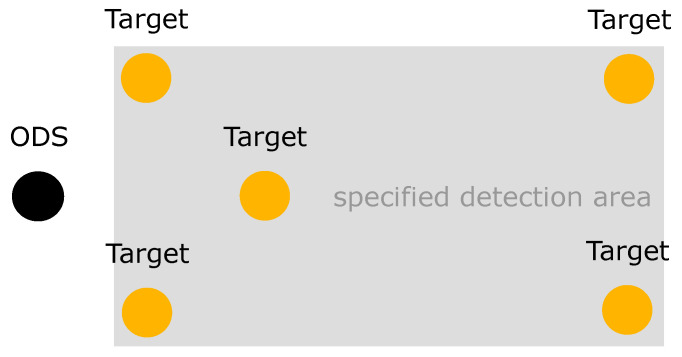
In the first test scenario, the ODS and test target are in a static state. The test target is in front of the ODS in their specified detection area (SDA).

Test category 1 is represented by the first test scenario. The test target is moved in front of the ODS into their specified detection area (SDA) (Figure 5). As soon as the ODS and the test target are in a static state, the ODSs are activated and a measurement is performed. The detection capability is tested both directly before the ODS and also at the edges of the SDA.

**Figure 6:**
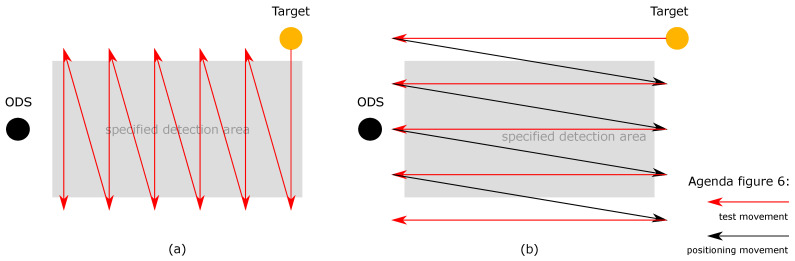
In the second test scenario, the ODS is in static state and the test target in dynamic state. The test scenario is divided in a lateral and longitudinal part: (**a**) In the lateral part, the test target is moved into the SDA from the left and right in a “zig-zag” movement from the ODS’s point of view; (**b**) in the longitudinal part, the test target is moved from front to back in a “zig-zag” movement into the SDA of the ODS from the point of view of the ODS.

The REDA of the second test category can be realized by the test scenario 2. The ODSs are in the static state and the test target is in the dynamic state. The test target is moved into the SDA from the left and right in a “zig-zag” movement from the ODS point of view (Figure 6a) and is then moved from front to back in a “zig-zag” movement into the SDA of the ODS from the point of view of the ODS (Figure 6b). This ensures that the SDA is passed from left and right, but also from front to back.

**Figure 7:**
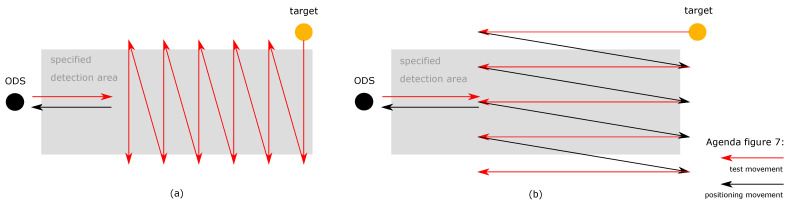
In the third test scenario, the ODS and the test target are in dynamic state. The test scenario is divided in a lateral and longitudinal part: (**a**) In the lateral part, the ODSs are moved forward and the test target is moved into the SDA from the left to right in “zig-zag” movement from the ODS point of view at the same time; (**b**) in the longitudinal part, the ODSs are moved forward and the test target is moved from back to front in a “zig-zag” movement into the SDA of the ODS from the point of view of the ODS. The illustrations show only a schematic representation. The size and length ratios can vary.

The REDA of the fourth test category can be realized by test scenario 3. The ODS and the test target are in dynamic state. The ODSs are moved forward. At the same time, the test target is moved into the SDA from the left to right in “zig-zag” movement from the ODS’s point of view (Figure 7a). Then, similar to test scenario 2, the test target is moved from back to front in a “zig-zag” movement into the SDA of the ODS from the point of view of the ODS (Figure 7b). This ensures that during the forward movement of the ODS, the SDA is passed through from left to right and from front to back.

**Figure 8:**
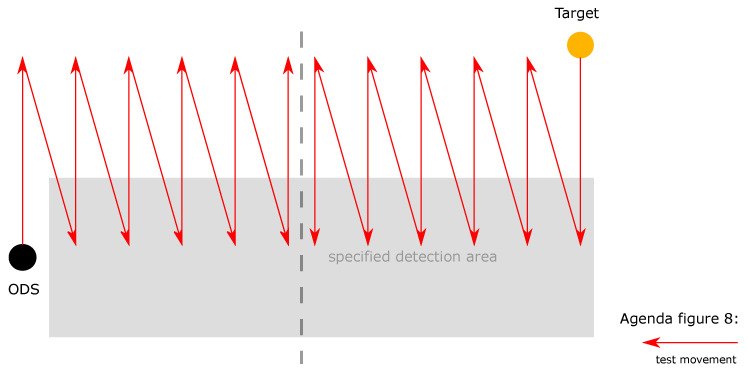
In the fourth test scenario the ODS and the test target are in dynamic state. The ODSs are moved from the left and right in a “zig-zag” movement and the test target is moved in the opposite direction to the ODS from right to left in a “zig-zag” movement from back to front.

For the fourth test category, in the fourth test scenario the ODS and the test target are in dynamic state. The ODS are moved from the left and right in a “zig-zag” movement and the test target is moved in the opposite direction to the ODS from right to left in a “zig-zag” movement from back to front (Figure 8). With this test the “worst-case” scenario is tested, when persons are running sideways into the SDA. The ODSs are accelerated sideways during cornering and a person runs into the SDA from the left or right.

Other scenario constellations with ODS and test target exist. Nevertheless, these scenarios are mostly different and are limited to the first characterization of the detection capability. The main goal of the test method is to determine the limits and sizes of the SDA of the ODS under different outdoor environmental conditions. Based on the results, the ODS can be pre-selected for the mobile machine under the tested environmental conditions. Further constellations arise with the individual locations of the ODS. They are checked during a direct validation on a mobile machine.

#### 2.5.2. Definition: Real Environment Detection Area (REDA)

A real environment detection area (REDA) can be created for an ODS in a specific environmental condition, regardless of the measurement principle used. For this purpose, a test target will be moved through the SDA of the ODS from the outside on the basis of the defined test scenarios. A fixed update rate will be used to record the positions of the ODS and test target. The relative distance between the ODS and the test target describes a test point in front of the ODS. For each test point, it is noted whether the ODS has detected the test target or not. In addition, the current environmental condition, the current positions of the ODS and the test object and their current speeds are recorded.

**Figure 9:**
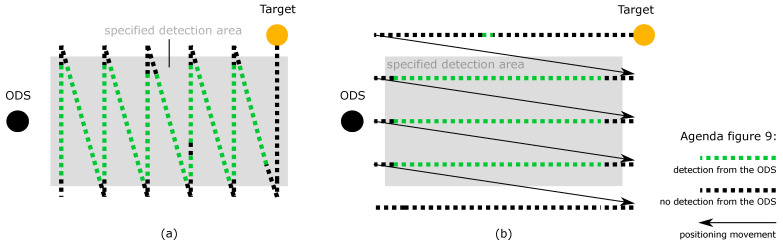
Records of the second test scenario: (**a**) The detections from the ODS are recorded with green dots when the test target enters laterally and passes through the SDA perpendicular to the sensor. No detections of the ODS are recorded as black dots. (**b**) The detections from the ODS are recorded with green dots during the longitudinal entry and passage of the test target through the SDA. No detections of the ODS are recorded as black dots.

In Figure 9, the record of an REDA is shown using the 2nd test scenario. Both parts of the test scenario are required to record a complete REDA. In Figure 9a, the detections are recorded when the test target enters laterally and passes through the SDA perpendicular to the ODS. In Figure 9b, the detections during the longitudinal entry and passage of the test target through the SDA are presented. The dotted lines represent the measuring point resolution. Green dots symbolize a detection of the ODS and black dots symbolize no detection of an ODS. Due to disturbances, it is possible that green dots are outside the SDA and black dots are within the SDA.

**Figure 10:**
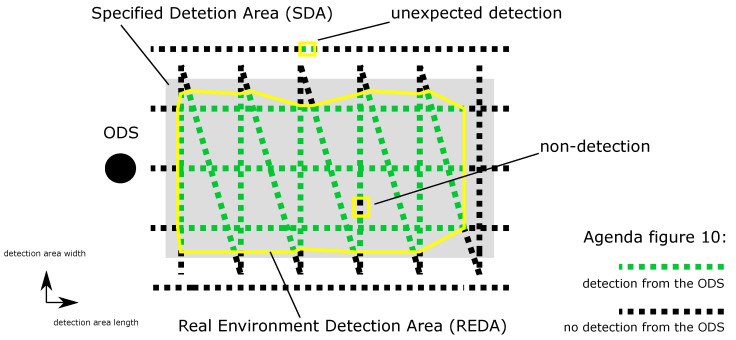
Superimposing the two records from the second test scenario and identifying a real environment detection area (REDA) for each ODS by a common contour (yellow marker). Non-detections and unexpected detections must be considered.

After recording is completed for both parts of the second test scenario, a first REDA (yellow marker) can be defined for each ODS in the current environmental condition in Figure 10. This is done by superimposing the two records and identifying an REDA for each ODS by a common contour. Non-detections and unexpected detections must be considered and their interpretation is explained in Section 2.5.4.

These REDAs are determined separately for each ODS in test scenarios 2 to 4. Section 2.5.3 describes the creation of REDAMs based on the REDAs, taking into account the different environmental conditions. In Section 2.5.4, the evaluation of the REDAs and REDAMs is explained.

#### 2.5.3. Definition: Real Environment Detection Area Matrix (REDAM)

**Figure 11:**
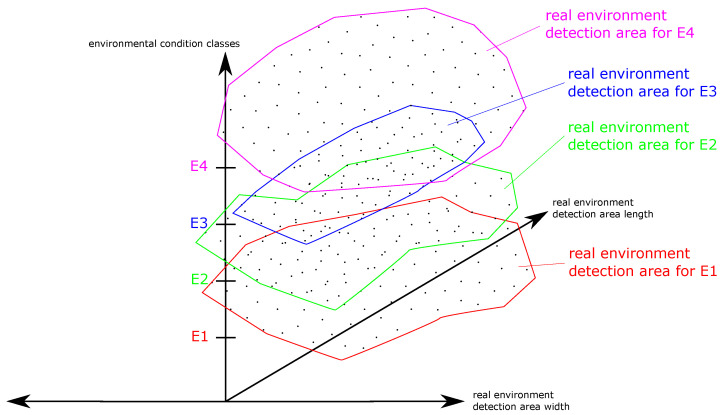
A real environment detection area matrix (REDAM) describes the detection capability of an ODS over all tested environmental conditions (E1–E4). All REDAs can be displayed comparably one above the other for evaluation.

A real environment detection area matrix (REDAM) describes the detection capability of an ODS over all tested environmental conditions. During the one-year long-term test, the REDAs of the ODS will be determined for all existing environmental conditions. In an REDAM, all REDAs of the ODS of the year can be displayed in a Cartesian coordinate system. An REDA is displayed along the abscissa and ordinate axes. On the application, the environmental conditions are summarized and displayed in classes (E1–E4). In this way all REDAs can be displayed comparably one above the other for evaluation along the applicate axis (Figure 11).

#### 2.5.4. Evaluation Procedure

When evaluating the REDAs, the areas have to be eliminated from false detections which are not directly visible in the fields. For this purpose, the positions of the detected object specified by the ODS will be recorded for each test point. This evaluation step is not performed for systems that cannot specify the position of the detected object. If the position of the detected object specified by the ODS does not match the actual position of the test target, a false detection is assumed. The next step is to investigate the positions of detections and non-detections in the REDA. In Figure 10, an REDA is shown which, from the ODS point of view, has an unexpected detection on the left outside the REDA. If the test target is not at the position of the unexpected detection, a false detection can be concluded. False detections are a safety risk, because a large number of false detections can lead to a higher risk of manipulation and thus to no safe operation. This environmental condition is therefore marked as safety-critical for the ODS and results in a gap in the availability under all existing environmental conditions.

In addition, Figure 10 shows a non-detection within the REDA. Non-detection within a safety area represents a safety risk. A safe operation is therefore not possible in spite of the REDA in this environmental condition class, because false and non-detections cannot be excluded. This environmental condition is also marked as safety-critical for the ODS and also results in a gap in the availability under all existing environmental conditions.

**Figure 12:**
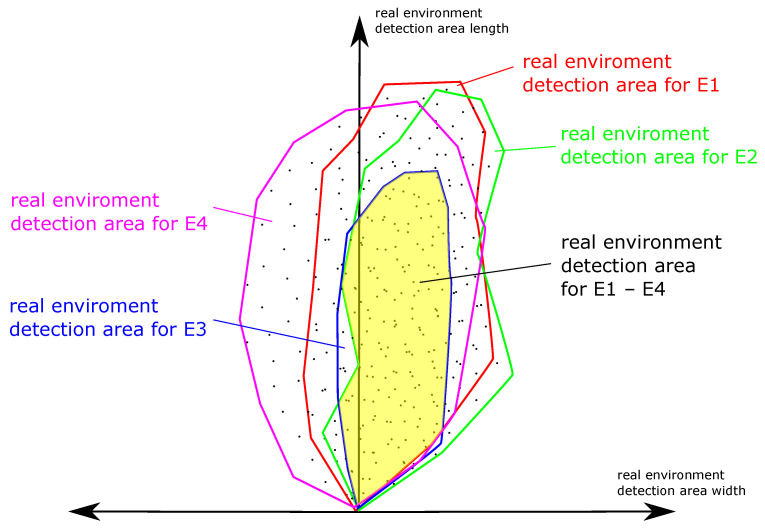
Evaluation of a real environment detection area matrix (REDAM): From the perspective from the top of the applicate axis, the REDA can be identified (yellow area) which results under all environmental conditions.

A REDAM is used to display all REDAs of one ODS. The matrix displaces the detection properties of an ODS in a common view under all non-safety-critical environmental conditions (see Figure 11). If the REDAs of the ODS are viewed from the perspective from the top of the applicate axis, the REDA can be identified (yellow area) which results under all environmental conditions (Figure 12). Environmental conditions that have been assessed as uncertain based on existing non-detection and false detection in the REDAs must not be taken into account. These uncertain environmental conditions must be excluded for the ODS.

Thus, an REDA can be defined for each ODS, which is valid for environmental conditions where no false- and non-detection occurred. As described before, the false- and non-detections result in gaps in the availability under all existing environmental conditions. An ODS fusion can be used to close this gaps. In an ODS fusion, the individual detection decisions of different ODSs are logically combined (decision methodology), resulting in one detection decision. Combining different REDAs of ODS in an REDAM, gaps can be closed and create an REDA, which includes all environmental conditions. This allows the REDAM to identify an optimal ODS fusion for all measured environmental conditions.

In the research project, an REDA resulting from an REDAM will be developed, which will be considered the relevant vehicle data (e.g., dimensions and speed) in addition to all measured environmental conditions. For further mobile machines, individual REDAs resulting from an REDAM can be created based on the presented test method. Here, the specific environmental conditions at the planned location as well as machine-specific data (e.g., dimensions and speed) can be taken into account. In this case, an OSD fusion may be necessary.

## 3. Result: Realization of the Test Stand

A new type of test stand was installed on a farm in order to practice the new test method in a long-term test. This test stand offers many technical possibilities to test the ODS and to determine their robustness and detection capability under different environmental conditions, thus enabling the individual selection of suitable technologies for the autonomous agricultural machines. As described above, the concept and this first test stand is adapted to the requirements of our application example, the autonomous feeding mixer. For the realization of the new test methods for further application examples, a test stand with other dimensions or a different installation site can also be selected.

The movement spaces are realized by means of two two-axis gantries of the company Bahr Modultechnik GmbH. Each two-axis gantry consists of three axes, which means that a total of six servo motors are used for the entire test stand. By the drive technology of the company Beckhoff Automation GmbH & Co. KG, the speeds specified in Section 2.3 are achieved with an acceleration of 8 $\frac{m}{s^{2}}$ and a positioning accuracy of ±1 mm. With the entire system technology from Beckhoff Automation GmbH & Co. KG, with the system a maximum update rate of 3 ms for the recording of the ODS data is achieved. Thus, at a speed of 2 $\frac{m}{s}$, a distance of 6 mm between two test points described in Section 2.5.2 can be realized.

Matching the drive technology, the test stand control is also equipped with components from Beckhoff Automation GmbH & Co. KG. In addition to the control of the servo motors, the test stand control communicates with all ODS and the weather station. Thus, the detection information of the ODS, the current positions and speed of the ODS and the test target, as well as the current environmental conditions can be bundled and stored in a database. Predefined test scenarios are performed by the test stand, which are automatically triggered depending on a time and environmental condition trigger. This means that if a change of the environmental conditions is measured via the weather station or a preset time is reached, a measurement is automatically performed based on the defined test scenarios. In addition, the test stand can be accessed remotely at any time and special measurements can be performed.

As shown in Figure 4, an extension of the Davis Vantage Pro 2 6163 EU weather station from Davis Instruments and the VISIC620 visibility measuring device from SICK AG is used. With the extended weather station and the visibility measuring device, the following environmental parameters are determined among others: Temperature, humidity, air pressure, precipitation (for rain, hail and snow), wind direction and speed, UV and solar radiation and visibility (for dust, fog and dew). With an outdoor camera with night vision function, an image of the test stand scenario can be recorded for the REDAs.

**Figure 13:**
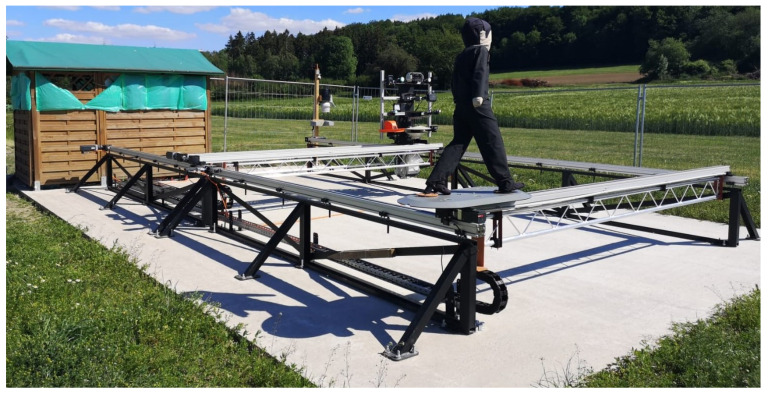
The figure shows the real test stand on an agricultural farm. In the foreground is the test target. Behind it the sensor holder is shown in the figure. Left of the sensor holder the extended weather station with the visibility measuring device and the camera is shown. In the upper left corner of the figure, a hut with the test stand control is shown.

In Figure 13, a current image of the test stand is displayed.

A total of 15 ODSs with 6 different sensor types are provided for the test stand by 8 different sensor manufacturers from industry and the automotive sector. The ODSs are divided into groups so that they cannot influence each other. In order to obtain an independent and fair test result, each ODS is parametrized independently by the manufacturer for the expected test scenarios and environmental conditions.

An existing test target from the automotive sector is used as test target. Compared to industry standards, it has a higher reflectivity for the NIR-wavelength range and is used for testing driver assistance systems for example. For this reason, the test target has been modified with a new material that its optical reflectivity properties also meet industry standards for driverless industrial trucks. The aging of the materials during outdoor use is checked and taken into account by spectral measurements. In a further study the effects of the new material on the radar reflectivity properties could be measured, no significant changes could be detected and the realistic reflectivity of the test target compared to humans could be confirmed.

In Section 2.5.1, the following questions have been defined. They will now be answered based on the developed test methods:

1. Can the ODS detect the test target under the current environmental conditions? With the first test scenario, static tests are performed to verify a general detection of the test target.
2. How large is the REDA of the ODS in static state under the current environmental conditions? In the second test scenario, the REDA of a static ODS was systematically traversed using the test target and a constant rate was used to determine whether the test target could be detected. Using the REDAs generated in this way, the size of the REDA of each ODS can be determined under the current environmental conditions in its static state.
3. Are there gaps and detection faults in the REDA of the ODS in the static state under the current environmental conditions? Using the REDAs from the second test scenario, gaps and detection faults in the REDAs can be determined for a static ODS.
4. How large is the REDA of the ODS in dynamic state under the current environmental conditions? With the third and fourth test scenario, the REDA of a dynamic ODS was systematically traversed using the test target and a constant rate was used to determine whether the test target could be detected. Using the REDAs generated in this way, the size of the REDA of each ODS can be determined under the current environmental conditions in the dynamic state.
5. Are there gaps and detection faults in the REDA of the ODS in the dynamic state under the current environmental conditions? Based on the REDAs from the third and fourth test scenario, gaps and detection faults in the REDA can be determined for a dynamic ODS.

## 4. Conclusions and Outlook

This article presents an important milestone and the next step in the development process of autonomous agriculture machines. As an impact of research into industry, within the research project “Agro-Safety”, a novel test method realized by a dynamic test stand is developed to test and compare the robustness and detection capability of commercially available ODS in a long-term test around the clock for 365 days a year and 24 h a day in continuous outdoor use for the very first time. A test over a longer period of time is needed to test as much as possible all occurring environmental conditions. This leads to the fact that it is a test that is determined by the naturally occurring environmental conditions. This corresponds to the reality of unpredictable/determinable environmental conditions in the field and makes the test method and test stand so unique. Thus, the new test method allows the individual selection of ODS for different autonomous mobile machines. For this purpose, the test stand can be adapted to the individual requirements of the application environment and the individual machine parameters. In this way, a test stand can also be adapted, for example, for plant production or other autonomous mobile working machines.

It has to be taken into account, during a given test period of one year, not all extreme weather conditions will occur. It must also be validated whether an ODS alone can guarantee sufficient availability with an acceptable level of safety under all environmental conditions, or whether a fusion of ODS is required for availability over all environmental conditions. In an ODS fusion, the individual detection decisions of different ODS are logically combined (decision methodology), resulting in one detection decision. In the future, these findings can be derived from the REDAs created by the test stand and the resulting REDAMs. If no or insufficient information is available for certain environmental conditions, this gap can be closed by continuously continuing outdoor tests or using the test stand in environmental simulation chambers. By the specific simulation of environmental conditions in the environmental simulation chamber, REDAs can be determined for environmental conditions that rarely occur in outdoor environments. It is also possible to repeat measurements for statistical evaluation of the detection capability of ODS.

A possible different positioning of the ODS on an autonomous mobile work machine but also possible worst-case scenarios in the working areas of the autonomous feeding mixer should be examined. For these reasons, a validation of the new human protection system directly on the vehicle is absolutely necessary after the selection of the ODS. The different application scenarios in the working areas of the autonomous feeding mixer must be validated. The test method and the test stand represent an abstract evaluation of the detection capability, but require a detailed validation on the application machine with its various environmental scenarios. In addition to the functional validation, a verification of the hardware structure and software implementation in the ODS must be conducted with the safety requirements of the autonomous work machine.

For the determination of ODS for use for human protection on autonomous, mobile work machines, the realistic “worst-case” simulation of humans by the test target must also be validated. For this purpose, a new material was used which meets the optical reflectivity properties of a standard for driverless industrial trucks. Nevertheless, the aging of the materials during outdoor use is checked and will taken into account by spectral measurements. These changes influence the test method and have to be considered. For this reason, a statistical evaluation of the test target is also planned. Likewise, possible changed parameters on the test stand are checked in further investigations and the effects must be considered in the data evaluation.

In further work, in addition to the detection information of each ODS, the corresponding raw data can also be collected. As described before, in an ODS fusion, the individual detection decisions of different ODS are logically combined (decision methodology), resulting in one detection decision with a better availability of the ODS. Another way to increase the availability of the ODS is a sensor fusion. In contrast to ODS fusion, sensor fusion fuses the recorded raw data of different ODS and resulting then in one detection decision. New and possibly better algorithms can be developed independently of the ODS using the measured raw data. Here, algorithms can be developed for general object detection as well as for special objects such as people, which are presented as humanoid test targets on the test stand. For the development of this algorithms, the test stand automatically generates exceptional information about the recorded raw data. On the one hand, the detection information of the ODS is available, on the other hand, the real position of the object is available. The detection decision of the ODS could be used as reference data for the verification of the newly developed algorithm, but could also be directly integrated into the decision making process. The recorded information, where the object is real located, can be used as data for verification of the newly developed algorithm or as information to label the record row data automatically. This labeled raw data could then be used as training data for neural networks or artificial intelligence in the newly developed algorithms.

## Figures and Tables

**Table 1:** Overview of pedestrian speeds and the classification of the applied test target speed.

Speed Source	Speed Value
male children aged 5 years (Kramer et al.) [18]:	3.51 $\frac{m}{s}$
running adult (Euro NCAP) [11]:	2.22 $\frac{m}{s}$
child aged 6–7 years (Euro NCAP) [11]:	1.38 $\frac{m}{s}$
adult (BS ISO 19237:2017-12-15) [16]:	1.38 $\frac{m}{s}$
child aged 6–7 years (project “Agro-Safety”):	2.3 $\frac{m}{s}$

## Abbreviations

The following abbreviations are used in this manuscript:

LiDAR	Light Detection And Ranging
NIR	Near-infrared
ODS	Object Detection System
REDA	Real Environment Detection Area
REDAM	Real Environment Detection Area Matrix
SDA	Specified Detection Area
ToF Camera	Time-of-Flight Camera
TÜV	German Technical Inspection Agency
